# Adapting Young Adults’ In-Shoe Motion Sensor Gait Models for Knee Evaluation in Older Adults: A Study on Osteoarthritis and Healthy Knees

**DOI:** 10.3390/s25072167

**Published:** 2025-03-28

**Authors:** Chenhui Huang, Kenichiro Fukushi, Haruki Yaguchi, Keita Honda, Yusuke Sekiguchi, Zhenwei Wang, Yoshitaka Nozaki, Kentaro Nakahara, Satoru Ebihara, Shin-Ichi Izumi

**Affiliations:** 1 Biometrics Research Labs, NEC Corporation, Hinode 1131, Abiko 270-1198, Chiba, Japan; 2 Department of Rehabilitation Medicine, Graduate School of Medicine, Tohoku University, 2-1 Seiryo-machi, Aoba-ku, Sendai 980-8575, Miyagi, Japan; 3 Department of Rehabilitation Medicine, Sendai Red Cross Hospital, 2-43-3 Honcho, Yagiyama, Sendai 982-8501, Miyagi, Japan; 4 Tsurumaki-Onsen Hospital, 1-16-1, Tsurumaki-kita, Hatano 257-0001, Kanagawa, Japan

**Keywords:** gait analysis, in-shoe motion sensor, foot motion, knee motion, stiff knee

## Abstract

The human knee joint is crucial for mobility, especially in older adults who are susceptible to conditions like osteoarthritis (OA). Traditionally, assessing knee health requires complex gait analysis in clinical settings, which limits opportunities for convenient and continuous monitoring. This study leverages advancements in wearable technology to explore the adaptation of models based on in-shoe motion sensors (IMS), initially trained on young adults, for evaluating knee function in older populations, both healthy and with OA. Data were collected from 44 older OA patients, presenting various levels of severity, and 20 healthy older adults, with a focus on key knee indicators: knee angle measures (S1 to S3), temporal gait parameters (S4 and S5), and knee angular jerk cost metrics (S6 to S8). The models effectively identified trends and differences across these indicators between the healthy group and the OA group. Notably, in indicators S1, S2, S3, S7, and S8, the models exhibited a large effect size in correlation with true values. These findings suggest that gait models derived from younger, healthy individuals are possible to be robustly adapted for non-invasive, everyday monitoring of knee health in older adults, offering valuable insights for the early detection and management of knee impairments. However, limitations such as fixed biases due to differences in measurement systems and sensor placement inaccuracies were identified. Future research will aim to enhance model precision by addressing these limitations through domain adaptation techniques and improved sensor calibration.

## 1. Introduction

### 1.1. Background

Daily locomotion in humans relies heavily on the proper functioning of the knee joint, which is essential for weight-bearing, balance, and overall mobility [1,2]. Impairments in knee function—whether due to injury or musculoskeletal conditions such as osteoarthritis (OA)—can markedly reduce quality of life, particularly among older adults, who are more vulnerable to such ailments [3]. Traditionally, gait analysis for assessing knee health has been confined to hospital settings or specialized facilities, limiting its utility for continuous, daily monitoring and early-phase rehabilitation.

Recent advancements in the Internet of Things (IoT) and wearable technologies have led to the development of smart devices capable of monitoring various aspects of human movement. Among these, in-shoe motion sensors (IMSs) have emerged as a promising tool for capturing foot kinematics in a non-invasive and user-friendly manner [4,5]. Prior studies have demonstrated that IMSs can accurately estimate a range of physical indicators—including foot function, muscle strength, balance, mobility, and even pelvic motion—by analyzing the motion of the foot during gait [6,7,8,9,10]. Although IMSs are not positioned directly at the knee, previous investigations have shown that foot motion data can reliably reflect knee joint behavior, thereby enabling the detection of gait features associated with knee OA [11,12]. This relationship is largely attributable to the biomechanical coupling within the lower limb, whereby alterations in foot movement correspond to changes in knee dynamics [13]. Consequently, we propose that IMS-based assessment represents a practical means for daily knee condition monitoring and early OA prevention.

Constructing robust models for knee motion assessment typically requires comprehensive data from both healthy individuals and OA patients across a wide range of ages. However, collecting extensive patient data is often time-consuming and resource-intensive. To address this challenge, we hypothesize that models developed from the gait data of young, healthy participants—whose gait patterns are relatively stable and less influenced by age-related or pathological factors—can be adapted to detect variations in knee behavior among both older healthy individuals and those with knee OA. This hypothesis is supported by seminal work in gait biomechanics, which demonstrates that the fundamental kinematic framework governing human gait is preserved across different populations [14]. Furthermore, research by Andriacchi et al. [15] indicates that key knee motion parameters maintain their biomechanical structure even in the presence of OA.

To operationalize this hypothesis, we focus on the variability in key gait parameters—differences that may reflect underlying biomechanical changes. Specifically, we define variability as the differences observed in knee angular excursions, temporal gait metrics (e.g., stance and swing durations), and angular jerk cost (AJC) indicators. In healthy individuals, these measures tend to be consistent; however, in OA patients, increased variability is observed due to factors like joint degeneration, pain-induced compensatory mechanisms, and diverse disease severities [16,17]. Hausdorff [18] further emphasized that differentiating between age-related and disease-related alterations in gait is crucial for developing effective clinical interventions.

In our previous study, we utilized IMS-measured foot motion during the gait of healthy young participants to develop lightweight estimation models for eight knee motion indicators [19]. These indicators—derived from the knee joint angle in the sagittal plane—encompass three types of knee angle, two temporal parameters [20,21,22,23,24,25], and three AJC measures [26]. The details of these indicators will be illustrated in Section 2.2. The previous biomechanical studies [20,21,22,23,24,25,26] indicated that these indicators are characteristic and effective for evaluating knee motion impairment. The models, constructed via multivariate linear regression, are efficient enough for implementation on edge devices. In the present study, we will directly apply these models to cohorts of older healthy individuals and knee OA patients. This approach will not only test the robustness of our models but also validate our hypothesis, ultimately advancing the use of IMS in everyday knee health assessment.

### 1.2. Main Contribution of This Study

We evaluated the feasibility of the preliminarily constructed models on both healthy and knee-OA older adults. Our results suggest that models derived from gait data of young, healthy individuals may have potential for assessing knee function in older adults, including those with knee OA. The sole-to-ground angle (SGA) signals obtained from IMS—which reflect foot posture in the inertial coordinate system—are of particular interest. Specifically, the SGA in the sagittal plane (*E_x_*) during walking is influenced not only by the ankle joint’s dorsiflexion/plantarflexion but also by the knee joint’s flexion/extension, making the predictors derived from the *E_x_* waveform valuable for further analysis. Consequently, this study also investigates the relationship between the SGA in the sagittal plane and knee impairment indicators across different participant groups.

## 2. Materials and Methods

### 2.1. Participant

In this study, we collected data from 44 older participants with knee OA (11 males and 33 females, denoted as Group OA) and 20 healthy older participants (12 males and 8 females, denoted as Group H) at Tohoku University Hospital. All participants were required to be capable of walking in daily life. Moreover, while no formal cognitive assessments were administered, each participant demonstrated sufficient cognitive ability to understand the study procedures and provide informed consent.

For knee OA patients, the inclusion criteria were as follows: (1) radiographic evidence of osteoarthritic changes in the knee, (2) knee pain localized exclusively to the knee, (3) the ability to walk in daily life, and (4) being an adult. For healthy adults, the inclusion criteria were simply the ability to walk in daily life and being an adult. Exclusion criteria for both groups were a history of any lesion or surgery affecting the lower limb or lumbar spine, neurological conditions impacting gait, visual field defects, and orthopedic or neurological disorders that could affect the measurements. Additionally, knee OA patients with pain in regions beyond the knee or with conditions (e.g., respiratory or circulatory diseases) that limit physical activity were excluded.

Knee OA severity was evaluated by an orthopedic surgeon using the Kellgren–Lawrence (KL) grading scale [27]. The sample size for this study was determined by including all eligible subjects recruited during the study period. All participants provided informed consent, and the study was approved by the Institutional Review Board of Tohoku University (Approval ID: 2022-1-1072).

The characteristics of the participants are described in Table 1, with no significant age difference observed between Group H and Group OA, while they were significantly older than the participants for model training. There was no significant difference in weight among three groups. A statistically significant difference in height was found between the training dataset and both Group H and Group OA.

### 2.2. Target Knee Indicators

**Figure 1:**
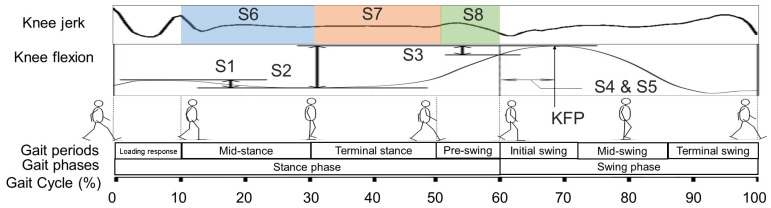
Selected knee motion indicators in one stride of knee flexion angle waveform (sagittal plane) modified from Ref. [19]. S1 to S3 depicts the knee angle indicators; S4 to S5 depicts temporal indicators; S6 to S8 depicts AJC indicators. Blocks with different color on the knee jerk waveform represent the region of interest in the gait cycle for calculating four types of AJC indicators. AJC: angular jerk cost; KFP: knee flexion peak.

Figure 1 illustrates the target knee indicators (S1 to S8) in this study. S1 represents the difference between the knee flexion angle values at 1 percentage gait cycle (%GC) and 30 %GC during the stance phase. During this period, knee flexion indicates the ability to cushion the impact after the heel strikes the ground. S2 represents the difference between the valley angle and the maximum knee flexion peak (KFP), reflecting the range of knee flexion as the lower limb transitions from the stance phase to the swing phase. S3 is the difference between the angle at toe-off and the maximum flexion angle, highlighting the range of knee flexion during the swing phase without ground support. S4 represents the duration, measured in seconds, from the toe-off to the KFP. S5 indicates the time duration of the same range as S4, expressed as a percentage of the swing phase, calculated using Equation (1),

(1) $$S5=\frac{t_{KFP}-t_{St}}{t_{Sw}}=\frac{S4}{t_{Sw}}$$

where *t*_KFP_ and *t_St_* represent the time duration from the start of one stride to KFP and toe-off, respectively. *t_St_* also signifies the stance phase’s time duration, while *t_Sw_* means the time duration of swing phase. The AJC is calculated by integrating the knee jerk waveform (Figure 1) and can be expressed by (2) [21],

(2) $$AJC=\log\int_{t_{1}}^{t_{2}}{\left(\frac{\Delta^{3}\theta }{\Delta t^{3}}\right)}^{2}dt$$

where *t* represents time, *t*_1_ and *t*_2_ denote the start and end of the region of interest gait period, and *θ* is the knee flexion angle during the gait. In Figure 1, blocks in different colors represent the region of interest gait period of the AJC, including mid-stance (S6), terminal stance (S7), and pre-swing (S8). Among these eight indicators, the first five reflect the static characteristics of knee motion at specific phases of the gait cycle, while the last three reflect the dynamic characteristics of knee motion during gait. Patients with osteoarthritis (OA) are generally observed to have smaller values for S1 to S3 and S6 to S8, along with longer durations for S4 and S5 [20,21,22,23,24,25,26].

### 2.3. The Details for Preliminarily Determined Predictors

**Figure 2:**
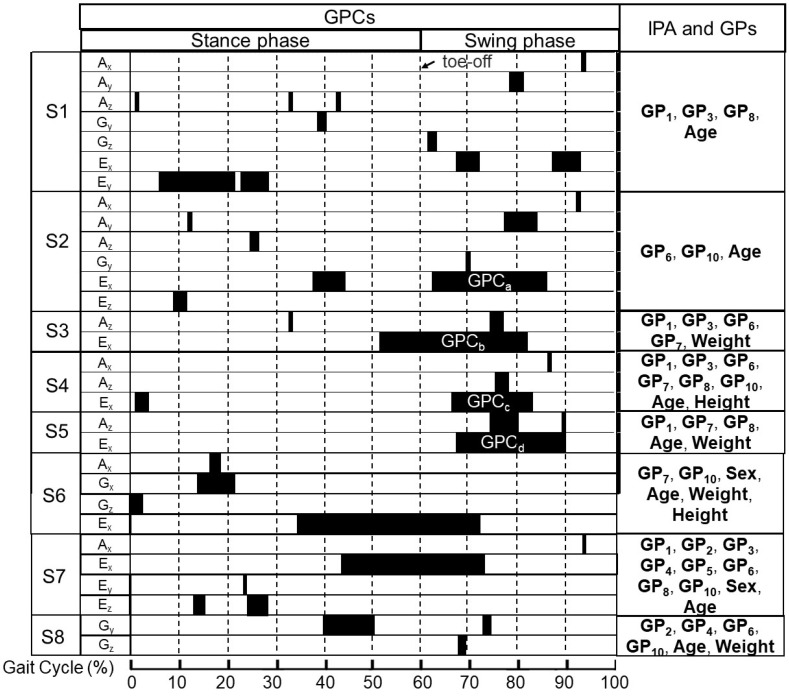
Schematic diagram of selected gait phase clusters (GPCs) and list of selected individual physical attributes (IPAs) and gait parameters (GPs) in each model according to the findings in our previous study [19]; black block: the range of GPC. Biomechanical direction: accelerations, *A_x_* (medial: +, lateral: −), *A_y_* (posterior: +, anterior: −), *A_z_* (superior: +, inferior: −)); angular velocities and sole-to-ground angles (SGAs), *G_x_*, *E_x_* (plantarflexion: +, dorsiflexion: −), *G_y_*, *E_y_* (eversion: +, inversion: −), and *G_z_*, *E_z_* (internal rotation: +, external rotation: −). GPC_a-d_: specific significant GPCs in SGA in plantarflexion direction during pre-swing to mid-swing, which will be discussed in the Results and Discussion sections.

The combinations of predictor and GPCs for S1 to S8 are shown in Figure 2. The models incorporated three types of predictors: (1) individual physical attributes (IPA), including sex (male: 0; female: 1), age, height, weight, and BMI; (2) 10 designed temporospatial gait parameters (GPs) measured by IMS [19], as described in Table 2; (3) the average amplitude of fragment of one cycle of nine-axis foot motion waveform during significant gait phases, where we denoted these gait phases as gait phase clusters (GPCs) and the average amplitudes as IMS predictors. Foot motion waveforms include three types of accelerations (*A_x_*, *A_y_*, and *A_z_*), three types of angular velocities (*G_x_*, *G_y_*, and *G_z_*), and three types of SGA waveforms (*E_x_*, *E_y_*, and *E_z_*).

### 2.4. Experiments

#### 2.4.1. Data Collection

**Figure 3:**
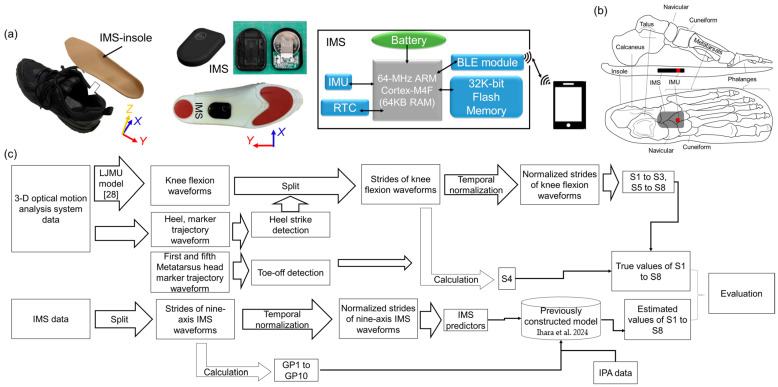
(**a**) Experimental Apparatus. The foot motion data were sent to the personal computer via Bluetooth transmission. The circuits of the in-shoe motion sensor (IMS), including a 6-axis IMU (BMI 160, Bosch Sensortec, Reutlingen, Germany), an ARM Cortex-M4F micro-control unit (MCU) (nRF52832, CPU: 64 MHz, RAM: 64 KB, ROM: 512 KB, Nordic Semiconductor, Oslo, Norway), an EEPROM (S-24C32C, 32K-bit, ABLIC, Tokyo, Japan), a real-time clock (RTC) (RX8130CE, EPSON, Suwa, Japan), and a 3-volt lithium-coin battery (CR2430, 300 mAh, Maxell, Tokyo, Japan). The MCU included a Bluetooth low-energy (BLE) module. (**b**) Location of the IMS in the shoe. (**c**) Brief flowchart of data processing and model validation flow. LJMU model: Liverpool John Moores University biomechanical model. Ihara et al., 2024 [19].

Participants were instructed to walk 8 m at their self-selected speed without using a cane. They performed five to seven trials in a laboratory walkway while wearing shoes equipped with IMS insoles. The schematic of the apparatus, the module inside the IMS, and the axes of the IMS signals are illustrated in Figure 3a. For participants with a foot size of 24 cm, for instance, the IMU inside the IMS was approximately located under the intermediate cuneiform (Figure 3b). The shoes fit tightly, modeling the mid-foot and hindfoot as a rigid body, thus allowing us to equate the IMS signals with foot motion signals. All participants wore the same type of sports shoes (GEL-KAYANO 24, ASICS, Kobe, Japan). During this feasibility study, detailed foot motion waveforms were recorded in real time by transferring them to a smartphone via the Bluetooth universal asynchronous receiver/transmitter (UART) mode (Figure 3a). Partial data loss occurred in the foot motion waveforms due to packet loss induced by limitations in the Bluetooth communication capacity and conditions. The sampling rate was set at 100 Hz.

Additionally, reflector markers were attached to their bodies according to the Liverpool John Moores University biomechanical model (LJMU model) [28] to measure the marker trajectories and kinematics during gait. For the calibration before the measurements, subjects were asked to stand in a predefined anatomical position (e.g., T-pose or neutral stance) while motion capture cameras recorded their posture and then perform predefined movements, such as hip and knee flexion, to allow the system to refine joint center estimations based on functional motion. This step established a reference frame for joint centers and segment orientations. During the gait trials, the trajectories of each marker in the LJMU model were directly measured using a 3-D optical motion analysis system (MAC 3D, Motion Analysis Corporation, Santa Rosa, CA, USA), while the subjects’ foot motions were simultaneously recorded with the IMS.

#### 2.4.2. Signal Processing

The flowchart of signal processing is shown in Figure 3c. All signal processing and data analysis were carried out using MATLAB (Version R2022b, MathWorks, Natick, MA, USA). Initially, the raw waveforms from the IMS were divided into individual strides by identifying heel strike events. These strides were then temporally normalized from 1 to 100% of the gait cycle (GC). Subsequently, the gait parameters (GPs) listed in Table 1, as well as the IMS predictors shown in Figure 2, were processed. Finally, the predictors were input into the previously constructed multivariate linear regression models [19], along with the IPA predictors, to estimate S1 to S8. It is important to note that stride length (GP_1_), maximum circumduction (GP_5_), and maximum foot height (GP_6_) were normalized by the participant’s height during the calculations.

The reference knee flexion angle data in this study were processed from the measured marker trajectories using Visual3D (Version Standard v6, C-Motion, Inc., Germantown, MD, USA). Including the knee flexion angle, all trajectory and kinematics data were partitioned within each stride by detecting heel strike events using the lowest point between two peaks in the trajectory of the markers on the heels. The timing of toe-off was detected by the lowest point in one stride of the average trajectory of the markers on the first and fifth metatarsus heads. Subsequently, we calculated the true values of knee indicators S1 to S8 for each stride. The data analyzed for this study were the averages of all steps within each trial, excluding the first and last steps.

#### 2.4.3. Result Analyses

At this stage, we firstly validated the models using all test data for Group H and OA, by applying Type (2, 1) intraclass correlation coefficient, denoted as ICC(2, 1), *r*, and the mean and standard deviation (SD) of true value (*M_t_* and *SD_t_*), estimated value (*M_e_* and *SD_e_*), and the difference between true and estimated value (*M_d_* and *SD_d_*). We tested the normality of S1 to S8 using the Kolmogorov–Smirnov test for Group H, Group OA, KL1-2, and KL3-4. The target variables in all groups followed the normal distribution. Secondly, we sought to assess whether the constructed models were able to successfully detect the differences between Group H and Group OA, as well as among the participants within Group H, participants with KL level 1 or 2, and participants with KL level 3 or 4. To classify participants in Group OA, we assigned them to the KL level corresponding to the more severe side of their limb. In the statistical analysis, *t*-tests and one-way ANOVA were utilized to compare differences between two groups and among three or more groups, respectively. The significance level was set at *p* < 0.05. For post hoc analysis of ANOVA, Tukey–Cramer correction was applied to adjust the *p*-values. The guidelines for interpreting the ICC inter-rater agreement were as follows: excellent (>0.750), good (0.600–0.750), fair (0.400–0.599), and poor (<0.400) [29]. The guidelines for interpreting *r* were as follows: none (<0.100), small (0.100 to 0.299), medium (0.300 to 0.499), and large (>0.499), and those for interpreting *R*^2^ were none (<0.020), small (0.020 to 0.129), medium (0.130 to 0.259), and large (>0.259) [26]. Additionally, the effect size of the difference between two groups was evaluated using Cohen’s *d*. The interpretation of *d* values followed these guidelines: none (< 0.200), small (0.200 to 0.499), medium (0.500 to 0.799), and large (> 0.799) [30].

## 3. Results

### 3.1. Gait Parameters in Different Groups

According to Table 3, except for GP_5_, the rest of the GPs indicated significant differences between healthy and OA participants. OA participants exhibited shorter strides, slower gait speed, weaker plantarflexion, dorsiflexion, decreased foot height, toe in/out angle, an extended stance phase, shortened swing phase, and lengthier stride time as compared to healthy participants.

### 3.2. Quantitative Evaluation of the Constructed Model

**Figure 4:**
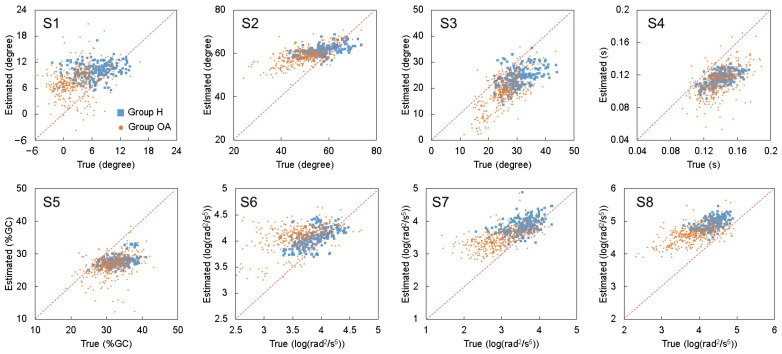
Agreement plots between true and estimated values of S1 to S8 of test data. The blue squares and the orange dots represent the data of Group H and Group OA, respectively. The red dashed lines represent the perfect agreement line.

The agreement plots of data gathered on Group H and OA are shown in Figure 4, and the quantitative metrics of model tests using all test data are depicted in Table 4. As a result, *SD_e_*s were larger than *SD_t_*s (Table 4), and fixed biases were observed in the data of Group H and OA between true and estimated value (Figure 4), which resulted in their low ICC values (Table 4).

Despite the possible biases between the wearable motion capture system and 3-D optical motion analysis system, by referencing the *r*s between true and estimated values, the correlation between true and estimated values of S2, S3, S4, S7, and S8 achieved large effect sizes, and that between true and estimated values of S1, S5, and S6 achieved medium effect sizes. These results indicated that the trends of indicators were well traced (Table 4).

According to *V* (the ratio of *SD_d_*/*SD_t_*) in Table 4, S3 and S4 achieved the best two *SD_d_*, which were only 0.86 and 0.65 times the value of *SD_t_*. Additionally, S8 also achieved a value of less than *SD_t_*. S6 did not achieve an ideal *SD_d_*, which was 1.51 times that of *SD_t_*. From Figure 4, we can confirm that S6 of Group H was relatively well estimated, while the agreement plots for Group OA were more scattered, which should be the reason for the higher *V*.

### 3.3. Comparison of Different Groups

**Figure 5:**
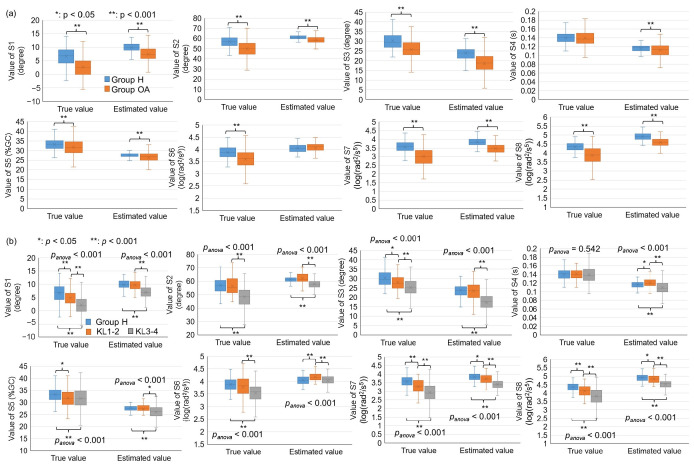
Comparison of true and estimated values and the statistics from S1 to S8 on Group H and Group OA. (**a**) Group H vs. Group OA; (**b**) Group H vs. KL1-2 vs. KL3-4. *p_anova_*: *p*-value of ANOVA among three groups. * and **: the corrected post hoc *p*-value of ANOVA below 0.05 and 0.001.

In this section, we applied the constructed models to a distinct group of participants, comprising 204 data points from Group H and 385 from Group OA, which included 76 KL1-2 data points and 309 KL3-4 data points. Relative to the actual value, in cases of indicators S1, S2, S6, S7, and S8, the estimated values for Group H, Group OA, KL1-2, and KL3-4 were all overestimated. Conversely, in the cases of S3, S4, and S5, the estimated values appeared underestimated (Figure 5). Despite these discrepancies, the constructed models successfully detected the trends and differences between Group H and Group OA when considering S1, S2, S3, S5, S7, and S8 (Figure 5a). Moreover, the models successfully identified the trends and differences across the three groups completely for S2, S7, and S8 and partially for S1 and S3 (Figure 5b). More details can be found in Supplementary Materials.

### 3.4. Connection Between Knee Motion Parameters and Foot Rotation in Sagittal Plane

**Figure 6:**
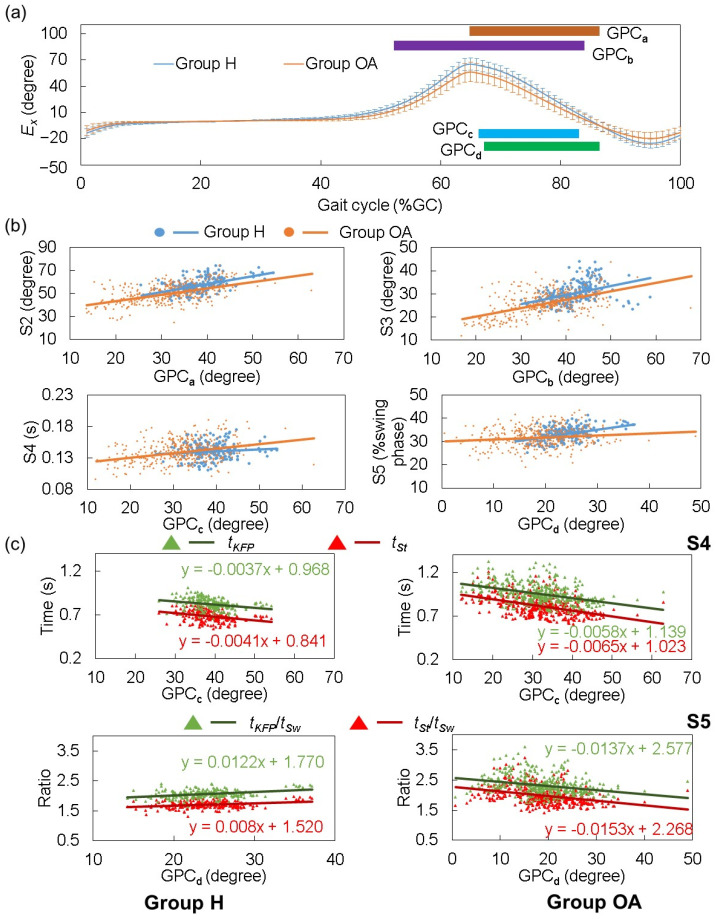
Analysis of the relationship between *E_x_* and stiff knee indicators. (**a**) Average and standard deviation of *E_x_* in different groups of subjects. The ROIs of corresponding GPCs (see Figure 3) are also represented by different color bars. (**b**) The scatter plots of stiff knee indicators S2 to S5 with their corresponding predictors come from *E_x_* in the different groups of subjects. (**c**) The scatter plots of GPC_c_ and GPC_d_ with the items in the formula for S4 and S5 calculation (see Equation (1)). The dots and the lines in different colors show the data and their tendencies. *t*_KFP_ and *t_St_*: the time duration from the start of one stride to knee flexion peak and toe-off, respectively. *t_Sw_*: the time duration of swing phase.

Figure 6a exhibits the *E_x_* waveforms across the various groups. That for Group OA was lower, particularly evident from the terminal phase to mid-swing. We analyzed the relation between each predictor and the corresponding indicator, reflected in Figure 6b. Across all S2 to S5 and in both groups, the indicator values increased in tandem with amplitude enhancement. The change in SGA predictors had a similar impact on S2 and S3 in both Group H and Group OA. However, for S4, the impact from the change in SGA was lower; for S5, the impact from the change in SGA was increased in Group H compared to Group OA.

S4 and S5 were measured as the difference between two items. Notably, an average delay of 0.13 s in the complete gait cycle was observed in Group OA relative to Group H (average *t*_KFP_ of Group OA and H: 0.95 s and 0.82 s). However, the toe-off event in Group OA also averaged a delay of approximately 0.13 s relative to Group H (average *t_St_* of Group OA and H: 0.81 s and 0.68 s), rendering the discrepancy in terms of the absolute time perspective of KFP to toe-off insignificant between groups. In contrast, when normalizing the duration by stride time, Group OA exhibited a shorter average KFP in %GC than Group H, of 11.5%GC and 13.1%GC, respectively. By observing *t*_KFP_/*t_St_*, i.e., KFP-swing phase ratio and *t_St_*/*t_Sw_*, i.e., stance-swing phase ratio, the respective values for Group OA were 0.25 and 0.26 larger than those for Group H.

Further examination of the relationship between GPC_c_ and GPC_d_ with elements constituting S4 and S5 is depicted in Figure 6c. In both S4 cases in Groups H and OA, both *t*_KFP_ and *t_St_* declined with the increase in GPC_c_; furthermore, the slope became steeper in the negative direction across these groups. Conversely, for the S5 cases, both *t*_KFP_ and *t_St_* increased with the elevation of GPC_d_ in Group H, while Group OA displayed the inverse tendency.

According to Equation (1), S4 is the difference between *t*_KFP_ and *t_St_*, and S5 is the difference between *t*_KFP_/*t_Sw_* and *t_St_*/*t_Sw_*. Concerning the first assumption, although GPC_c_ and GPC_d_ indeed weakened along the sequence of Groups H and OA, against our expectations, positive correlations were observed between S4 and S5 and both GPC_c_ and GPC_d_ in both groups. To address this contradiction, analysis was conducted as shown in Figure 6c. The results demonstrated that the regression line slope for *t*_KFP_ and *t*_KFP_/*t_Sw_* was larger than for *t_St_* and *t_St_*/*t_Sw_*, respectively, which accounted for the contradiction. Contrary to the second assumption in this study, no significant difference was observed between Group H and Group OA for S4. Moreover, S5 for Group OA was significantly shorter than that for Group H, a discrepancy from the earlier suggestions based on the stiff knee gait of participants with cerebral palsy [19]. By observing the results in Figure 6c, we found that for Group H, the slope of *t*_KFP_-GPC_c_ was 10% less than that of *t_St_*-GPC_c_, while in Group OA, the former was 11% less than the latter, where the slope differences (the impact of the change in GPC_c_) of both were almost the same. For S5, in Group H, the slope of *t*_KFP_/*t_Sw_*-GPC_d_ was 53% larger than that of *t_St_*/*t_Sw_*-GPC_d_, while in Group OA, the former was only 10% larger than the latter. Our IMS can directly detect *t_St_* and *t_St_*/*t_Sw_*. These findings demonstrate that rather than focusing on p4 and p5, focusing on the difference between the slopes of *t_St_*-GPC_c_ and *t*_KFP_/*t_Sw_*-GPC_d_ may hold more potential for the early detection of knee deterioration.

Furthermore, all groups displayed a medium to large, effect-sized, positive correlation between gait velocity and the peak of *E_x_* in the plantarflexion direction (*r* = 0.583, 0.454, 0.720). Upon analyzing the linear regression coefficient between gait velocity and *t*_KFP_, *t_St_*, and *t_Sw_*, we found that in Group H, *t*_KFP_, *t_St_*, and *t_Sw_* all showed a distinct downward trend. However, in Group OA, despite *t*_KFP_ and *t_St_* exhibiting a greater reduction trend than the other two groups, *t_Sw_* largely remained steady with increased gait velocity (Table 5). These observations suggest that different physical conditions may show variations in gait patterns. Specifically, increased gait velocity seems to impact both the stance and swing phase duration in healthy participants, whereas in participants with OA, it primarily manifests itself as a reduction in the proportion of the gait cycle spent in the stance phase.

## 4. Discussion

### 4.1. Connection Between Knee and Other Foot Motion Parameters

Participants with knee OA tend to have stiff knees [31], a condition adversely affecting gait. Goldberg et al. [21] and Ezaki et al. [32] have suggested that participants with stiff knee tendencies have lower S2 and longer S4 and S5 durations., i.e., late appearance of KFP during swing phase. In S2, we observed a clear correlation indicating that older individuals with deteriorating knees exhibit decreased knee flexion and *E_x_* during both the initial and mid-swing phases. This observation is in alignment with findings from earlier studies (Figure 6b). Deasy et al. [33] postulated that participants suffering from knee OA typically have weaker hip flexors, resulting in reduced elevation of the lower limb during the swing phase. Based on this understanding, we can infer that participants with reduced lower limb elevation during the swing phase likely have elongated S4 and S5 timings. Furthermore, Goldberg et al. [21] and Ezaki et al. [32] suggested that participants with worse knee motion performance may have longer S4 and S5. Yaguchi et al. [11] indicated that individuals with deteriorating knees exhibit decreased knee flexion and *E_x_* peak during swing phase. Therefore, we may assume that S4 and S5 should be (1) negatively correlated with GPC_c_ and GPC_d_ in both groups; and (2) longer in the order of Group OA and H, as we also discussed in our previous study [19]. In our previous study, we discussed the correlation among healthy younger participants, while in this study, we continued the discussion on this point among older healthy and OA participants to provide a deeper insight. The mean values of distinct segments of *E_x_*, from terminal stance to mid-swing, were specifically selected as principal predictors for the estimation of indicators S2 to S5. We denoted these segments as GPC_a_, GPC_b_, GPC_c_, and GPC_d_ (see Figure 3). As such, it is crucial to understand the relationship between *E_x_* and these indicators across different groups, which forms the focus of Section 3.4.

In our previous study [19], we also analyzed the characteristics of the knee impairment indicators and the relationship between SGA predictors and the indicators in the group of younger healthy participants, as shown in Table 4 and Figure 6. Comparing the previous and current studies, we observed a transition in mean values and gradients among all three groups showing that gait performance is better in the order of younger healthy, older healthy, and older OA groups. Notably, when compared to the healthy group, we detected a reversal in the gradient of the KFP-swing phase ratio and stance-swing phase ratio relative to SGA in Group OA. These findings underscore the nonlinear influence of age and OA on gait patterns, which likely contributed to the discrepancy observed between model-estimated values and true values in this study. However, we did not focus on how to model and compensate the nonlinear effects of age and OA on the constructed models in this study.

### 4.2. Limitation of This Technology

Despite the advancements made in this study, there are several limitations inherent to our methodology that warrant discussion. One of the primary concerns, as indicated by the low intraclass correlation coefficient (ICC) values observed in Figure 4, is the fixed biases between the true and estimated values for both the healthy older adults and those with osteoarthritis. These low ICC values can be attributed to the differences in measurement systems employed during the model’s development and the current study’s execution. Initially, knee motion data were captured using a wearable motion capture system equipped with multiple inertial measurement units (IMUs) on various segments of the lower limbs [19]. In contrast, the reference data for this study were gathered using a 3D optical motion analysis system that tracks marker trajectories on the body.

These disparities between systems introduce distinct challenges. The models and calibration processes for the two systems differ significantly. Sensor-embedded pants, for instance, use IMU signals (acceleration and angular velocity) to calculate the knee motion and calibrate the measurement using specific motion sequences [34], where each participant will be asked to adopt two postures: leaning forward with their hands pressed against a wall and standing upright. However, the calculation and calibration of the 3D motion analysis system relied on spatial position signals in the inertial coordinates. This fundamental difference can result in variations in baseline knee joint angle waveforms. Moreover, the event detection methodologies for identifying heel strikes and toe-offs also vary. Sensor-embedded pants rely on algorithms to estimate ground contact probabilities [35], contrasting with the 3D motion analysis system, which identifies these events more directly through marker–ground distance measurements. Such methodological discrepancies might lead to offsets in measurements, notably affecting variables like S4 and S5.

Another issue arises from the limited range of pants sizes available, which often resulted in improper fits for some participants. This misfit could compromise the precise positioning of the IMUs, affecting the accuracy of kinematic data collection and potentially introducing random noise into the recorded signals. These non-technical factors may contribute to the observed differences between true and estimated values.

In response to these challenges, it is crucial to emphasize that our model’s primary aim is not to achieve absolute agreement between estimated and true values but to effectively capture the trends and relative differences in knee impairment indicators, especially between differing participant groups. Despite the low ICC values, we observed strong Pearson’s correlation coefficients for several indicators, indicating that the model adeptly tracks meaningful variations in knee function. We also evaluated other key metrics, such as effect sizes and the ratio of standard deviations, to illustrate the model’s capacity to distinguish group differences and capture gait-related changes, thereby supporting its scientific validity.

Furthermore, acknowledging the systematic biases introduced by differing measurement methodologies, future work should involve exploring domain adaptation techniques [36] to better harmonize outputs across these systems. This approach could involve treating different measurement methods and varying participant conditions as distinct domains, ultimately enhancing model agreement while preserving its ability to detect significant trends. By addressing these considerations, we can improve the versatility of the constructed models and advance toward more precise monitoring and assessment of knee health.

## 5. Conclusions

In this study, based on tests of knee impairment indicators in older adult healthy and OA participants, the pretrained models from younger healthy participants successfully determined trends and differences in all three selected knee joint angle indicators, in one temporal indicator, and in two AJC indicators between Group H and Group OA. Moreover, even across all three groups, the models partially or completely identified trends and differences for all three selected knee joint angle indicators and two AJC indicators. We demonstrated the potential to estimate knee behaviors in older healthy and OA participants using only foot motion data captured by a single IMS and models constructed from younger healthy individuals. These breakthroughs may facilitate simplified daily monitoring of knee conditions.

Looking forward, we intend to improve the quantitative estimation capacity and versatility of the constructed models. We also aim to create an application to assist with user rehabilitation grounded in the insights derived from this study.

## Figures and Tables

**Table 1:** The characteristics of participants.

		Mean ± SD	
	Participants for Training [19]	Group H	Group OA
Number (M/F)	66 (35/31)	20 (12/8)	44 (11/33)
Number (KL1–2/KL3–4)	–	–	10/34
Age (years)	44.5 ± 15.9	69.0 ± 8.0 *	70.8 ± 9.4 *
Height (cm)	164.5 ± 9.3	161.0 ± 7.9 *	155.6 ± 9.7 *
Weight (kg)	61.4 ± 14.1	56.4 ± 11.0	63.0 ± 13.4

SD: standard deviation; *: indicates a statistically significant difference (*p* < 0.05) in height compared to the training data.

**Table 2:** The results for gait parameters depicted in Figure 2.

Gait Parameters	Description
GP_1_ (m)	Stride length
GP_2_ (m/s)	Gait speed
GP_3_ (°)	Maximum SGA in dorsiflexion direction
GP_4_ (°)	Maximum SGA in plantarflexion direction
GP_5_ (cm)	Maximum circumduction during swing phase
GP_6_ (cm)	Maximum foot height during swing phase
GP_7_ (°)	Toe in/out angle in the transverse plane
GP_8_ (%GC)	Proportion of stance phase
GP_9_ (%GC)	Proportion of stance phase
GP_10_ (s)	Stride time

%GC: percentage gait cycle.

**Table 3:** The gait performances of participants in different groups.

	Mean ± SD	
	Group H	Group OA
Data number (KL1-2/KL3-4)	204	385 (79/306)
GP_1_ (m)	1.32 ± 0.14	1.06 ± 0.22 *
GP_2_ (m/s)	1.24 ± 0.17	0.88 ± 0.22 *
GP_3_ (°)	27.13 ± 5.27	21.51 ± 7.09 *
GP_4_ (°)	64.34 ± 6.81	56.40 ± 10.70 *
GP_5_ (cm)	2.27 ± 0.91	2.37 ± 1.58
GP_6_ (cm)	13.72 ± 1.60	11.14 ± 2.57 *
GP_7_ (°)	11.27 ± 8.26	8.72 ± 8.39 *
GP_8_ (%GC)	63.04 ± 1.68	66.10 ± 3.10 *
GP_9_ (%GC)	36.96 ± 1.68	33.90 ± 3.10 *
GP_10_ (s)	1.08 ± 0.09	1.23 ± 0.13 *

Standard deviation (SD), percentage gait cycle (%GC), stride length (GP_1_), gait speed (GP_2_), maximum sole-to-ground angle (SGA) in dorsiflexion direction (GP_3_), SGA in plantarflexion direction (GP_4_), maximum circumduction (GP_5_), maximum foot height (GP_6_), and toe in/out angle in the transverse plane (GP_7_), proportion of stance phase (GP_8_) and swing phase (GP_9_), stride time (GP_10_); *: significance level *p* < 0.05 between Group H and OA.

**Table 4:** The model test results for S1 to S8.

	S1	S2	S3	S4	S5	S6	S7	S8
ICC	0.225	0.305	0.395	0.214	0.169	0.230	0.437	0.253
*r*	0.399	0.670	0.689	0.603	0.380	0.474	0.689	0.691
*M_t_*	8.31	59.19	27.14	0.128	30.49	3.97	3.71	4.63
*SD_t_*	3.17	5.10	5.26	0.016	3.59	0.24	0.34	0.37
*M_e_*	5.09	54.37	22.18	0.125	28.97	3.83	3.23	4.24
*SD_e_*	4.16	7.70	6.71	0.022	4.76	0.41	0.50	0.54
*M_d_*	4.24	7.08	−6.93	−0.024	−5.45	2.55	1.21	0.41
*SD_d_*	3.93	6.40	4.52	0.010	3.93	4.61	0.44	0.36
*V*	1.24	1.25	0.86	0.65	1.09	1.51	1.20	0.97

ICC: type (2, 1) of the intra-class correlation coefficient between true and estimated values; *r*: Pearson’s correlation coefficient between true and estimated values. *M_t_*: mean values of the true values; *SD_t_*: standard deviations of the true values. *M_e_*: mean values of the estimated values; *SD_e_*: standard deviations of the estimated values. *M_d_*: mean values of the difference between true and estimated values; *SD_d_*: standard deviations of the difference of true and estimated values. Units of *M_t_*, *SD_t_*, *M_d_* and *SD_d_*: degree (S1, S2, S3); s (S4); %swing phase (S5); log(rad^2^/s^5^) (S6 to S8). *V*: the ratio of *SD_d_*/*SD.*

**Table 5:** Linear coefficients of correlation of regressed line between gait velocity and *t*_KFP_, *t_St_*, and *t_Sw._*

	*t*_KFP_	*t_St_*
Group H	−0.250	−0.249
Group OA	−0.345	−0.366

## Data Availability

The datasets presented in this article are not readily available due to privacy, legal, or ethical reasons.

## Supplementary Materials

The following supporting information can be downloaded at: https://www.mdpi.com/article/10.3390/s25072167/s1, Table S1: The mean and standard deviation values of S1 to S8 in different groups.; Table S2: Cohen’s *d*s of Group H vs. Group OA, Group H vs KL1-2, Group H vs. KL3/4, and KL1/2 vs KL3-4.
