# Supplementary material for: Adapting Young Adults’ In-Shoe Motion Sensor Gait Models for Knee Evaluation in Older Adults: A Study on Osteoarthritis and Healthy Knees
Source: Sensors (Basel). 2025 Mar 28;25(7):2167. doi: 10.3390/s25072167 (PMC11991446; doi:10.3390/s25072167)
Supplement: Supplementary file 1 [file sensors-25-02167-s001.zip › sensors-3507696-supplementary.pdf]

Supplementary Materials

1. Details on comparison of knee motion indicator S1 to S8 between different groups

In this study, we evaluated the feasibility of the preliminarily constructed models for knee motion indicators S1 to S8 (see Figure 1 in the main manuscript) on both healthy and knee-OA older adults.

Table S1 demonstrated the true and estimated values in detail groups, which connects Figure 4 in the main text.

Table S1. The mean and standard deviation values of S1 to S8 in different groups.

	**Group H**				**Group OA**				**KL 1/2**				**KL 3/4**			
	**True**		**Estimated**		**True**		**Estimated**		**True**		**Estimated**		**True**		**Estimated**	
	M_t	SD_t	M_e	SD_e	M_t	SD_t	M_e	SD_e	M_t	SD_t	M_e	SD_e	M_t	SD_t	M_e	SD_e
**S1**	6.74	3.43	9.88	1.84	2.67	3.51	7.51	3.24	4.85	3.79	9.68	3.11	2.13	3.22	6.97	3.05
**S2**	57.08	6.19	61.29	2.24	50.06	8.15	58.69	3.88	56.49	6.77	62.21	3.52	48.40	7.64	57.78	3.43
**S3**	30.35	4.64	23.94	3.65	25.79	5.20	18.57	6.09	27.98	4.86	23.41	5.33	25.23	5.14	17.32	5.64
**S4**	0.140	0.013	0.116	0.008	0.139	0.017	0.112	0.017	0.141	0.015	0.121	0.014	0.139	0.018	0.110	0.017
**S5**	33.23	2.93	27.75	1.48	31.69	4.13	26.25	3.68	31.76	3.79	27.32	3.39	31.67	4.22	25.98	3.70
**S6**	3.88	0.25	4.05	0.18	3.59	0.41	4.07	0.23	3.78	0.44	4.18	0.16	3.54	0.39	4.04	0.24
**S7**	3.57	0.36	3.85	0.24	3.00	0.53	3.47	0.31	3.28	0.42	3.74	0.31	2.92	0.54	3.40	0.27
**S8**	4.35	0.26	4.92	0.18	3.87	0.50	4.60	0.28	4.11	0.38	4.84	0.25	3.81	0.51	4.54	0.25

Units: degree (S1, S2, S3); s (S4); %swing phase (S5); $log(rad^2/s^5)$ (S6 to S8). M_t: mean values of the true values; SD_t: standard deviations of the true values. M_e: mean values of the estimated values; SD_e: standard deviations of the estimated values. M_d: mean values of the difference between true and estimated values; SD_d: standard deviations of the difference of true and estimated values. Units of M_t, SD_t, M_d and SD_d: degree (S1, S2, S3); s (S4); %swing phase (S5); $log(rad^2/s^5)$ (S6 to S8).

2. Cohen's *d*s of Group H vs Group OA, Group H vs KL1-2, Group H vs KL3/4, and KL1/2 vs KL3-4

We also tested whether the previously model can detect the trends of S1 to S8 between different groups of participants (see Figure 5 in the main text). Besides, further analysis was performed using Cohen's *d*s for those indicators where the model successfully detected trends and differences (Table S2). The true S1, S2, S3, S7, and S8 values demonstrated a large effect size, while S5 showed a small effect size between Group H and Group OA. All estimated values for the successful indicators achieved the same level of effect size as their true counterparts, except for S2. Among the three groups, barring S2, the estimated values for the other successful cases achieved no lower level than their true counterparts. These findings suggest that models crafted from the data of younger, healthy participants could potentially be applicable to older, healthy individuals as well as older participants with osteoarthritis.

Table S2. Cohen's *d*s of Group H vs Group OA, Group H vs KL1-2, Group H vs KL3/4, and KL1/2 vs KL3-4.

	d_0		d_1		d_2		d_3	
	d_{t0}	d_{e0}	d_{t1}	d_{e1}	d_{t2}	d_{e2}	d_{t3}	d_{e3}
**S1**	**1.167**	**0.835**	*0.534*	0.087	**1.393**	**1.105**	**0.815**	**0.884**
**S2**	**0.930**	*0.533*	0.088	0.266	**1.220**	*0.775*	**1.083**	**1.284**
**S3**	**0.909**	**1.001**	*0.503*	0.125	**1.035**	**1.341**	*0.541*	**1.091**
**S4**	0.048	0.309	0.057	0.440	0.072	0.483	0.111	*0.687*

**S5**	0.411	0.486	0.459	0.199	0.416	*0.588*	0.024	0.367
**S6**	**0.804**	0.075	0.314	*0.729*	**1.010**	0.061	*0.608*	*0.629*
**S7**	**1.187**	**1.316**	*0.768*	0.421	**1.360**	**1.737**	*0.683*	**1.217**
**S8**	**1.107**	**1.308**	*0.793*	0.413	**1.268**	**1.731**	*0.625*	**1.224**

d_0: Group H vs Group OA (Figure 5a); d_1: Group H vs KL1-2 (Figure 5b); d_2: Group H vs KL3/4 (Figure 5b); d_3: KL1/2 vs KL3-4 (Figure 5b); d_t: true value; d_e: estimated value. Texts in black: $d < 0.200$ (none). Underlined text: $0.200 \le d < 0.500$ (small effect size). Italic texts: $0.500 \le d < 0.800$ (medium effect size). Bolded texts: $d \ge -0.800$ (large effect size).
